# Adipocyte differentiation between obese and lean conditions depends on changes in miRNA expression

**DOI:** 10.1038/s41598-022-15331-2

**Published:** 2022-07-07

**Authors:** Yerim Heo, Hyunjung Kim, Jiwon Lim, Sun Shim Choi

**Affiliations:** Division of Biomedical Convergence, College of Biomedical Science, Institute of Bioscience & Biotechnology, Kangwon National University, Chuncheon, 24341 Korea

**Keywords:** Computational biology and bioinformatics, Biomarkers, Diseases

## Abstract

Adipogenesis is the process by which precursor cells, preadipocytes (preACs), differentiate into adipocytes (ACs). Here, we investigated differentially expressed miRNAs (DEMs) between the two conditions to understand the regulatory role of miRNAs in altering adipogenesis-related mRNAs. A total of 812 and 748 DEMs were obtained in lean and obese conditions, respectively. The up- and downregulated DEMs were highly concordant with each other in both lean and obese conditions; however, DEMs related to adipogenesis in obese conditions were more strongly downregulated than DEMs related to adipogenesis in lean conditions. There were more obese-specific downregulated DEMs than lean-specific downregulated DEMs; in contrast, there were more lean-specific upregulated DEMs than obese-specific upregulated DEMs. Approximately 45% of DEMs were mapped to the list of miRNA-target mRNA pairs when DEMs were matched to the experimentally validated list of miRNA-target mRNA information of miRTarBase. Many of the target mRNAs were differentially expressed genes (DEGs) with functions in processes such as inflammatory responses and fat metabolism. In particular, a total of 25 miRNAs that target three upregulated adipogenesis-associated inflammatory genes (*IL-6*, *TNF-α*, and *IL-1β*) were commonly altered during adipogenesis. Taken together, our study reveals the types of adipogenesis-related miRNAs that are altered and the degree to which they influence healthy or pathogenic adipogenesis.

## Introduction

Adipogenesis is the process by which mesenchymal-driven precursors, i.e., preadipocytes (preACs), differentiate into adipocytes (ACs) that can store excessive energy as lipids^1–3^. Dysregulated miRNAs are known to accelerate or inhibit adipogenesis^2,4^. MicroRNAs (miRNAs), a class of small noncoding RNAs, are well-established posttranscriptional regulators that generally downregulate gene expression by degrading or destabilizing target mRNAs or by lowering the efficiency of target mRNA translations^5–11^. MiRNAs are implicated as important players in various biological processes, including cell proliferation, differentiation, embryonic development, and cell fate determination^4,11–14^. In addition, many recent studies have shown that several human diseases, such as cancers, cardiovascular diseases, and type 2 diabetes, are associated with abnormal alterations in the expression of miRNAs^2,15–18^. Obesity is also a well-known pathogenic physiological condition involving alterations in the expression of various miRNAs^19–21^.

Since the functional role of miRNAs in fat cell differentiation was first recognized by miR-14 in *Drosophila*, where miR-14 deletion caused enlarged lipid droplets in adipocytes^2,22,23^, numerous miRNAs have been reported to be altered during adipogenesis or obesity. Studies have reported unique sets of miRNAs by comparing gene expression between preACs and ACs or by comparing gene expression of lean and obese adipose tissues^24–30^; the former was designed to identify miRNAs involved in adipogenesis, while the latter was to identify miRNAs that represent the difference between lean and obese ACs. For instance, Klöting et al.^29^ identified 11 miRNAs in humans that were differentially expressed in different fat adipose tissues (i.e., omental adipose tissues in lean and obese individuals), including miR-17-5p, miR-132, miR-134, miR-181a, miR-27a, miR-30e, miR-140, miR-155, miR-210, miR-147 and miR-197, of which miR-155 and miR-210 were expressed at higher levels in samples from obese subjects than in samples from lean subjects. Martinelli et al.^30^ found that miR-519d, i.e., a miRNA upregulated during adipocyte differentiation, was overexpressed in subcutaneous adipose tissues (SATs) from obese subjects compared to SATs from lean subjects. According to Ortega et al.^31^, miR-221, miR-424, miR-210 and miR-503 were downregulated, whereas miR-30c and miR-378 were highly upregulated during adipogenesis. Skarn et al.^32^ showed that miR-155, miR-221, and miR-222 inhibited adipogenesis by targeting *PPARG* and *CEBPA*.

It is worth noting that studies have often linked mRNAs and miRNAs that are altered in adipogenesis to obesity, although adipogenesis is a process that occurs in both lean and obese conditions. In other words, many studies have identified molecular changes, such as changes in mRNA or miRNA expression levels, under the assumption that molecular changes involved in adipogenesis are linked to changes that occur in the development of obesity. However, Xie et al.^25^ found that genes upregulated during adipogenesis were not necessarily upregulated in obese conditions by showing that upregulated adipogenesis-associated miRNAs were actually downregulated in obese states or vice versa.

In our previous study, we examined differential gene expression separately for adipogenesis in lean conditions (L_Ag) and adipogenesis in obese conditions (O_Ag) using RNA-seq data derived from purified ACs and preACs of human visceral adipose tissues (VATs) of lean and obese individuals^33^. In that study, we reported a surprising observation that inflammatory genes, i.e., well-known upregulated genes associated with obesity, were expressed at lower levels in ACs from obese subjects than in ACs from lean subjects. We also showed that inflammatory genes such as *IL-6*, *TNF-α*, and *IL-1β* were significantly upregulated during adipogenesis in both lean and obese conditions (i.e., *[AC]*_*e*_ > *[preAC]*_*e*_, when the expression levels of genes (represented by *[]*_e_) were compared between AC and preAC); however, the fold changes (FCs) in the upregulations of those genes were significantly greater for lean conditions than for obese conditions^33^. Overall, it was concluded that the unexpected observation mentioned above was because L_Ag requires higher upregulation of inflammatory genes than O_Ag. In that study, we thus proposed the idea that inflammatory genes may have a positive role in adipogenesis in lean or healthy individuals.

In the present work, we attempt to explore what epigenetic regulators are involved in controlling the expression of these inflammatory genes. For this, we isolated miRNAs that target and control mRNAs previously identified as differentially expressed genes (DEGs), including inflammatory genes described above. We wondered how the adipogenesis-associated miRNAs identified under lean conditions, i.e., differentially expressed miRNAs (DEMs) of L_Ag, differed from those identified in obese conditions. In addition, we searched for miRNAs that are common regulators of adipogenesis that play roles in both lean and obese conditions and identified the miRNAs that are involved in selectively controlling either O_Ag or L_Ag.

## Results

### Identification of DEMs in ACs compared to preACs

**Figure 1:**
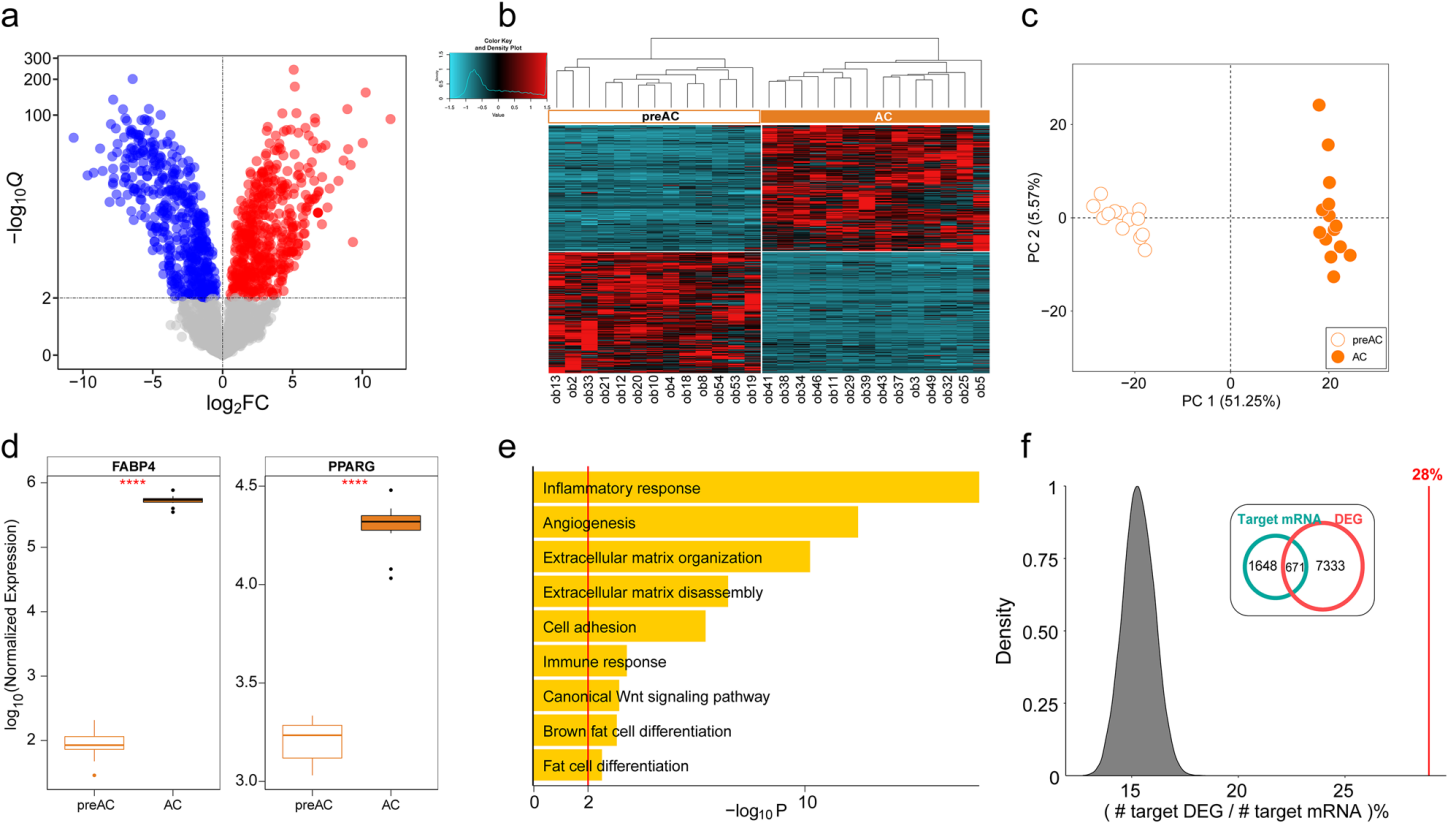
Detection of miRNAs controlling adipogenesis. (**a**) Volcano plot representing the differential expression of miRNAs in ACs compared to preACs. − *log*_*10*_*Q* values (Y-axis) were plotted against *log*_*2*_FC values (X-axis). Significantly upregulated miRNAs (*Q*-values < 0.01, FC > 0) are shown as red points, while significantly downregulated miRNAs (*Q*-values < 0.01, FC < 0) are shown as blue points. (**b**) Heatmap depicting patterns of Z score scaled expression for DEMs along with unsupervised hierarchical clustering. The rows of the heatmap are the 929 DEMs were selected according to the threshold, *Q*-value < 0.01, sorted by unsupervised hierarchical clustering based on their gene expression profiles. The columns of the heatmap are the 13 preACs and 14 ACs, also sorted by unsupervised hierarchical clustering based on their gene expression profiles. (**c**) PCA of DEMs obtained from preAC and AC samples. DEMs selected according to the threshold, *Q*-value < 0.01, were used. (**d**) Boxplots illustrating the expression levels of the adipogenesis marker genes *FABP4* and *PPARG*. The DESeq2-normalized count of each gene was used as the expression level. ‘****’ denotes *P value* < 0.0001, DESeq2. (**e**) GO analysis of DEGs targeted by DEMs with *Q*-value < 0.01. The top GO (biological process) terms significantly enriched are shown in the bar graph. – *log*_*10*_*P* (X-axis) against each functional term (Y-axis) are depicted as yellow bars, and the red line annotates significance at a *P value* of 0.01 (− *log*_*10*_*P* = 2). (**f**) Distribution of the percentages (the number of DEGs in the DEM target genes) estimated during 10,000 random permutations and *(inset)* Venn diagram showing the number of DEM target genes and DEGs. For each permutation, 2319 genes (the number of DEM target genes) were randomly selected from all protein-coding genes, and the percentage of DEGs in the randomly selected genes was calculated. The red line indicates the observed percentage of DEGs in the miRNA target genes (28%).

In our previous study, we identified DEGs altered in expression levels during adipogenesis by comparing the gene expression of ACs and preACs^33^. Here, we aimed to investigate upstream miRNA perturbations to control those DEGs. For this purpose, we first identified differentially expressed miRNAs (i.e., DEMs) using the miRNA information produced by the miRNA-seq technique by comparing miRNA expression between 14 AC and 13 preAC samples and named them as Ag_DEMs (i.e., adipogenesis-associated DEMs). Note that the samples were subsets of the purified AC and preAC samples used for generating total RNA-seq data in Lee et al.^33^. A total of 929 miRNAs were determined to be altered during adipogenesis by a threshold of Q < 0.01 (Additional files 1, 2: Tables S1 and S2); these included 473 upregulated and 456 downregulated miRNAs (Fig. 1a). As shown in the heatmap (Fig. 1b) and principal component analysis (PCA) plot (Fig. 1c), the DEMs clustered AC and preAC samples separately, indicating that the expression of these miRNAs was significantly altered during the process of differentiation from preAC to AC. We also confirmed that two adipogenesis marker genes, *FABP4* and *PPARG*, were significantly upregulated in ACs compared to preACs (Fig. 1d). Subsequently, to determine whether those DEMs are possibly involved in controlling the expression of genes associated with adipogenesis, we estimated target mRNAs for the DEMs by overlapping the list of DEMs with miRNA-target mRNA pair information in miRTarBase. A total of 2,319 mRNAs were identified as target mRNAs controlled by the up- or downregulated DEMs (i.e., predicted DEM-target mRNAs), and then investigated for the functional categories to which these genes are allocated using gene ontology (GO) analysis. Interestingly, the GO functional terms assigned to those target mRNAs were the very functions that numerous studies, including our previous study, have repeatedly reported to be associated with obesity, including the inflammatory response, angiogenesis, extracellular matrix organization, cell adhesion, and fat cell differentiation (Fig. 1e). Furthermore, 28% of the predicted DEM-target mRNAs were actually identified as DEGs, and we demonstrated that this percentage could not be accounted for by random chance (Fig. 1f), given that only an average of 15.32% were identified as DEGs when genes were randomly selected as many as the number of the predicted DEM-target mRNAs during 10,000 iterations. We also confirmed that the primary functional classes of DEGs linked to DEMs were all related to functional terms associated with obesity described in Fig. 1e (Additional file 3: Fig. S1).

### Identification of DEMs involved in adipogenesis in lean and obese conditions

We next determined how different AC and preAC samples derived from lean and obese individuals were in terms of miRNA expression. As shown in Fig. S2a (Additional file 3: Fig. S2a), preACs and ACs had distinct miRNA expression profiles, and PC1 clearly differentiated preACs (represented by open circles) and ACs (represented by closed circles). However, both preAC samples and AC samples were not discriminated by PC1 clearly between lean and obese conditions (Additional file 3: Fig. S2a), which suggests that both lean and obese individuals have very similar miRNA expression profiles in preAC and AC samples.

**Figure 2:**
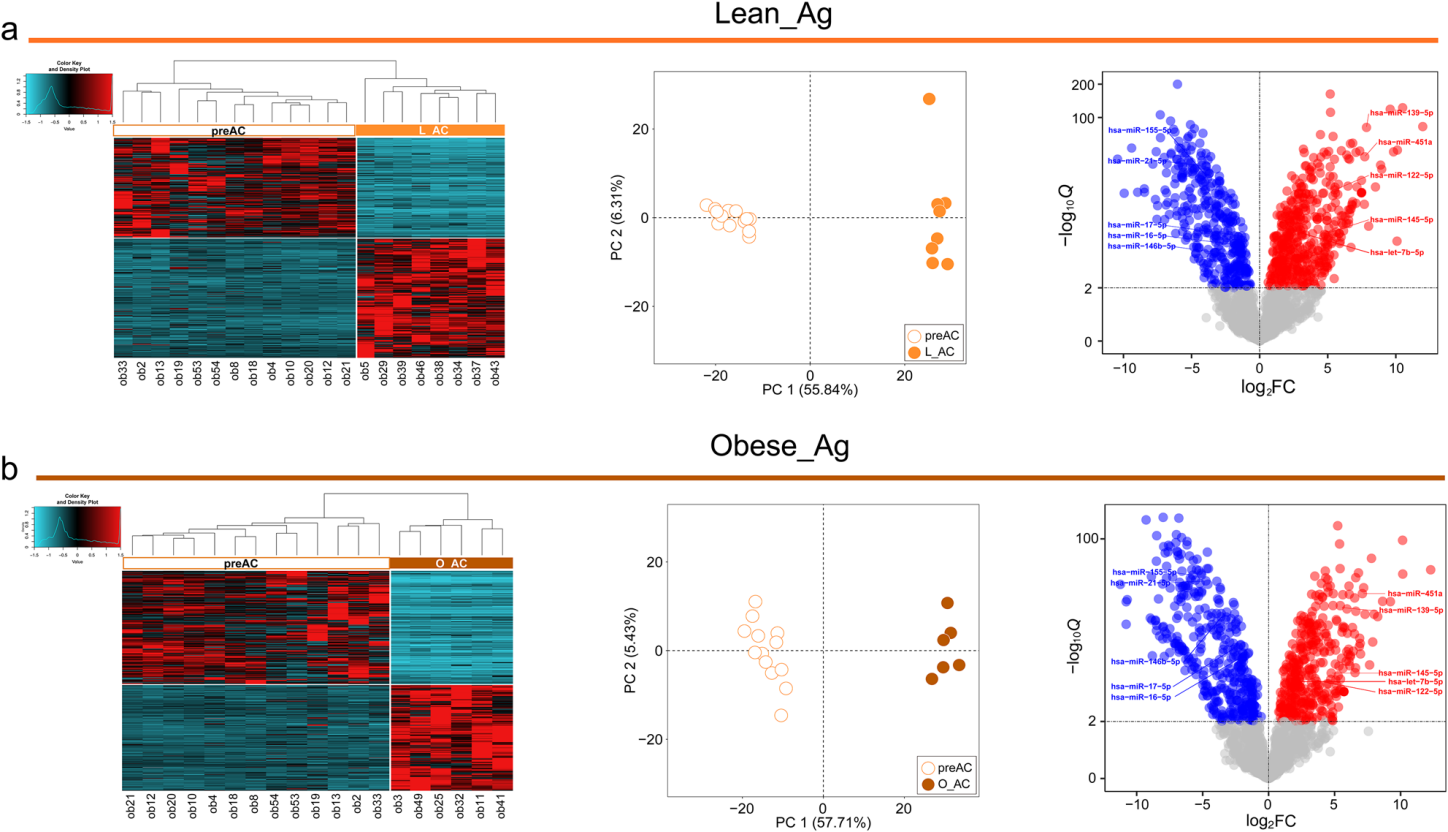
Expression patterns of DEMs detected in L_Ag and O_Ag. (**a**) *(left)* Heatmap for Z score scaled expression patterns of DEMs identified in L_Ag, *(middle)* PCA of preACs and L_ACs, *(right)* Volcano plot representing the differential expression of miRNAs in L_ACs compared to preACs. The details of the heatmap, PCA plot, and volcano plot are the same as in Fig. 1a–c, respectively. (**b**) *(left)* Same as the *left* panel of (**a**) but for O_Ag, *(middle)* Same as the *middle* panel of (**a**) but for preACs and O_ACs, *(right)* Same as the *right* panel of (**a**) but for preACs and O_ACs.

We next attempted to identify adipogenesis-associated DEMs for lean and obese conditions; the preAC samples clustered together in the PCA plot were used as a common control to estimate L_Ag-associated DEMs and O_Ag-associated DEMs. A total of 812 (443 upregulated and 369 downregulated) and 748 (362 upregulated and 386 downregulated) miRNAs were identified as L_Ag-associated DEMs and O_Ag-associated DEMs, respectively, using a threshold of Q < 0.01 (Additional file 3: Fig. S2b). Heatmaps, PCA plots, and volcano plots of DEMs are presented in Fig. 2a for L_Ag-associated DEMs and Fig. 2b for O_Ag-associated DEMs. The number of DEMs was similar between the two sets; however, a larger proportion of upregulated DEMs (54.5% upregulated *vs.* 45.5% downregulated) was observed for L_Ag, while a larger proportion of downregulated DEMs (48.4% upregulated *vs*. 51.6% downregulated) was observed for O_Ag.

### DEMs are highly concordant in adipogenesis between lean and obese conditions but differ in the magnitudes of expression FC

We have previously shown that the magnitude and direction of alterations in adipogenesis-related gene expression are highly similar in L_Ag and O_Ag, producing a *Pearson* correlation coefficient (*r*) of approximately 0.96^33^. We wondered whether such a high correlation coefficient between lean and obese conditions was maintained for miRNA expression.

**Figure 3:**
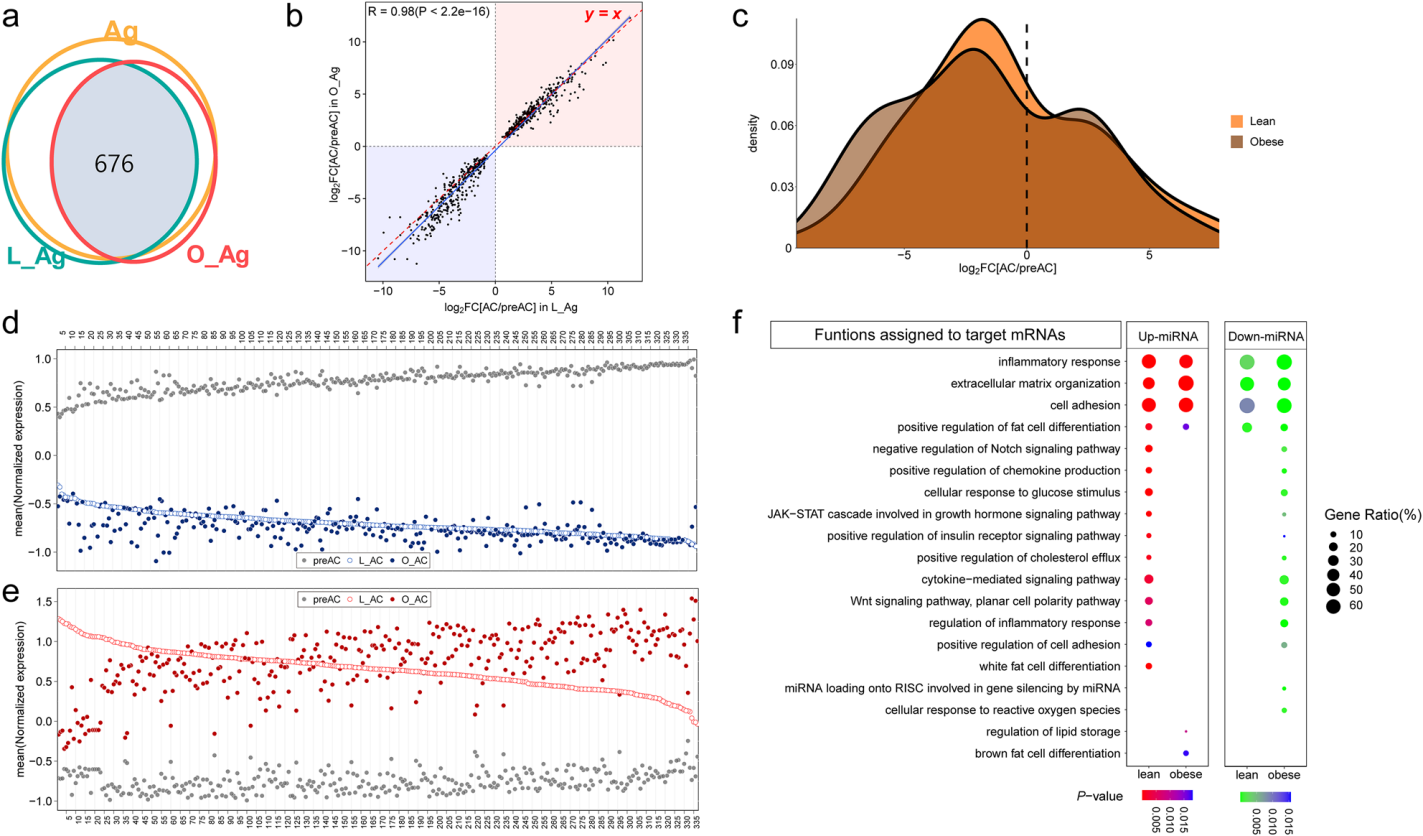
Analysis of DEMs commonly associated with both L_Ag and O_Ag. (**a**) Venn diagram depicting the number of common DEMs (*Q* < 0.01 in both L_Ag and O_Ag). The yellow circle represents the Ag_DEMs, and the green and red circles represent the L_Ag-associated DEMs and O_Ag-associated DEMs, respectively. The light blue shaded region represents the overlapping area between each comparison group. (**b**) Scatter plot for common DEMs. The upregulated and downregulated DEMs are shaded red and blue, respectively. *Log*_*2*_*FC* values of miRNA expression changes in L_Ag (X-axis) were plotted against those in O_Ag (Y-axis). The red dashed line indicates the line of equality (Y = X). The regression line between the values is shown in blue, and the gray shadow reflects the 95% confidence interval. **c.** Density plot of *Log*_*2*_*FCs* of common DEMs; density of miRNAs in L_Ag (orange) and in O_Ag (brown). (**d**) Dot plot for the mean DESeq2-normalized expression level of the commonly downregulated DEMs (*n* = 339). (**e**) Dot plot for the mean DESeq2-normalized expression level of the commonly upregulated DEMs (*n* = 337). (**d**, **e**) The numbers on the X-axis correspond to the commonly downregulated DEMs and upregulated DEMs (only multiples of 5 are shown), respectively. The DEM information corresponding to each number on the X-axis is given in Additional files 4 and 5 (Tables S3 and S4). (**f**) Dot plot of the GO functional terms in which genes targeted by common DEMs for each comparison group are enriched. ‘Lean’ and ‘obese’ on the X-axis indicate genes targeted by DEMs assigned to ‘Lean_Strong’ and ‘Obese_Strong’, respectively.

We found that a total of 676 DEMs corresponding to ~ 83% of L_Ag-associated DEMs and ~ 90% of O_Ag-associated DEMs were common DEMs (Fig. 3a; Additional files 4 and 5: Tables S3 and S4). Note that the number of Ag_DEMs represented by the yellow circle in Fig. 3a has a slight inconsistency with the numbers of L_Ag-associated DEMs and O_Ag-associated DEMs. In addition, the common DEMs were highly concordant between L_Ag and O_Ag, resulting in a *Pearson* correlation coefficient of ~ 0.98 for their FCs (Fig. 3b). Interestingly, the y = x line (red) closely coincided with the regression line (blue) for upregulated genes, whereas the two lines deviated slightly from each other for downregulated genes (Fig. 3b). This result indicates that the magnitude of FCs of downregulated DEMs may play an important role in differentiating between L_Ag and O_Ag, which is also confirmed by the density plot analysis in Fig. 3c, which shows very little difference in density between L_Ag and O_Ag for upregulated DEMs compared to downregulated DEMs. We next confirmed this observation at the single miRNA level by examining how each common DEM changed differently in L_Ag and O_Ag, respectively (Fig. 3d,e); a higher number of downregulated DEMs had lower expression levels in ACs from obese individuals (O_ACs, represented by the blue closed circle) than in ACs from lean individuals (L_ACs, represented by the blue open circle) (Fig. 3d), whereas the number of upregulated DEMs was similar between DEMs with higher expression levels in O_ACs than in L_ACs and DEMs with higher expression levels in L_Ag than in O_Ag (Fig. 3e). A scatter plot of FC values for each common DEM between L_Ag and O_Ag also led to the same conclusion (Additional file 3: Fig. S3).

To determine whether the differences in the magnitude of adipogenesis-associated FCs of miRNAs between lean and obese conditions are related to obesity, we categorized common DEMs into four groups: (i) ‘Lean_Strong_Up’: common DEMs that were more highly upregulated in L_Ag than O_Ag; (ii) ‘Lean_Strong_Down’: common DEMs that were more strongly downregulated in L_Ag than O_Ag; (iii) ‘Obese_Strong_Up’: common DEMs that were more highly upregulated in O_Ag than L_Ag; and (iv) ‘Obese_Strong_Down’: common DEMs that were more strongly downregulated in O_Ag than L_Ag. Interestingly, GO analysis showed that some obesity-associated functional terms, such as the inflammatory response, the extracellular matrix, and cell adhesion, appeared to be affected by both upregulated miRNAs and downregulated miRNAs identified from the L_Ag and O_Ag, whereas other functional terms, such as the *Notch* signaling pathway, response to glucose stimulus, white fat cell differentiation, and the *Wnt* signaling pathway, were influenced by the Lean_Strong_Up or by Obese_Strong_Down categories of DEMs (Fig. 3f).

### Identification of DEMs specifically involved in L_Ag or O_Ag

**Figure 4:**
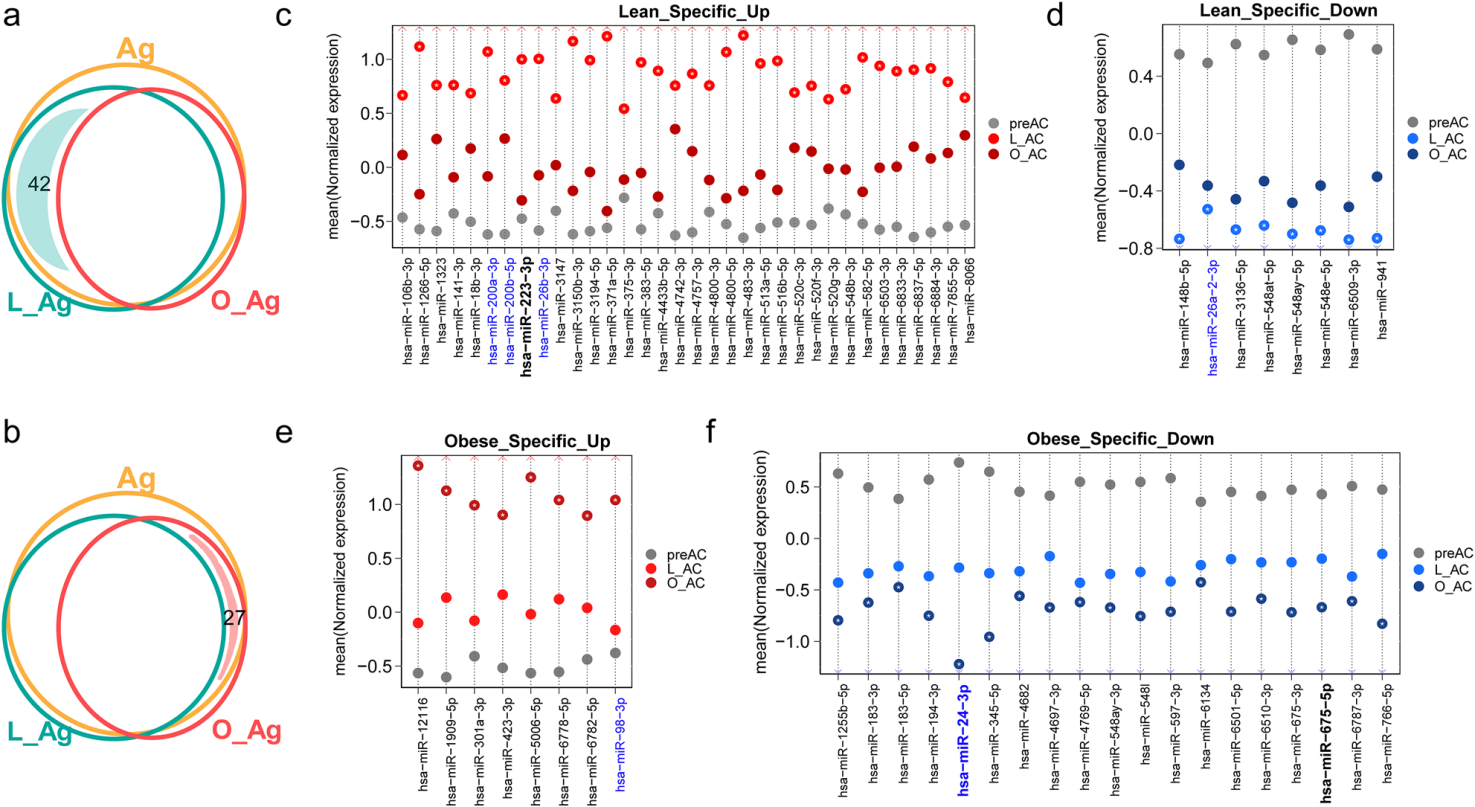
Analysis of DEMs identified only in the ‘Lean_Specific’ and ‘Obese_Specific’ categories. (**a**) Venn diagram depicting the number of DEMs obtained from the Lean_Specific category (*Q*-value < 0.01 in L_Ag but *Q*-value ≥ 0.05 in O_Ag). Refer to the legend of Fig. 3a for the yellow, green, and red circles. The light-green shaded region represents the L_Ag area overlapping with Ag but not with O_Ag. (**b**) Venn diagram depicting the number of DEMs obtained from the Obese_Specific category (*Q*-value < 0.01 in O_Ag but *Q*-value ≥ 0.05 in L_Ag). Note that the same meanings for the yellow, green, and red circles are the same as in (**a**). The light-red shaded region represents the O_Ag area overlapping with Ag but not with L_Ag. (**c**, **d**). The mean values (Y-axis) of miRNA expression are plotted for each cell type group. The miRNAs known to be associated with adipogenesis are colored blue, those associated with the inflammatory response are highlighted in bold, and those related to adipogenesis and the inflammatory response are colored blue and highlighted in bold. (**e**,** f**) Same as (**c**, **d**) but for DEMs obtained from the Obese_Specific category.

We next attempted to examine miRNAs that were DEMs only for the L_Ag condition or for the O_Ag condition to identify miRNAs that were considered to be associated with ‘L_Ag-specific’ or ‘O_Ag-specific’ downstream gene expression perturbations. For this purpose, the list of DEMs identified by a threshold of Q < 0.01 in one condition, either L_Ag or O_Ag, overlapped with the list of miRNAs excluded in DEMs by a threshold of Q ≥ 0.05 in another condition. As a result, we found that a total of 42 miRNAs (Fig. 4a) and 27 miRNAs (Fig. 4b) belonged to the ‘Lean_Specific’ and ‘Obese_Specific’ categories, respectively. Figure 4c and d are the plots representing each miRNA level grouped into ‘Lean_Specific_Up’ and ‘Lean_Specific_Down’ categories, respectively, containing DEMs that are specifically upregulated and downregulated, respectively, only for L_Ag. In contrast, Fig. 4e and f are the plots representing each miRNA level grouped into ‘Obese_Specific_Up’ and ‘Obese_Specific_Down’ categories, respectively, containing DEMs that are specifically upregulated and downregulated, respectively, only for O_Ag. Notably, the Lean_Specific category had a larger number of upregulated miRNAs than downregulated miRNAs (Fig. 4c vs. d), whereas the Obese_Specific category had a smaller number of upregulated miRNAs than downregulated miRNAs (Fig. 4e vs. f).

Some of the miRNAs grouped into the Lean_Specific_Up and Lean_Specific_Down categories, including miR-200a-3p, miR-200b-5p, and miR-26b-3p (upregulated) (Fig. 4c)^24,31,34,35^ and miR-26a-2-3p (downregulated) (Fig. 4d)^24,31^ are already known to be involved in adipogenesis. Some of the miRNAs in the Obese_Specific_Up and Obese_Specific_Down categories also overlapped with previously known adipogenesis-associated miRNAs, i.e., miR-98-3p in the Obese_Specific_Up (Fig. 4e) and miR-24-3p in the Obese_Specific_Down category (Fig. 4f)^31,36^. In particular, miR-223-3p in the Lean_Specific_Up category and miR-675-5p in the Obese_Specific_Down category (Fig. 4f) are among the previously known miRNAs involved in inflammatory response gene regulation^37–39^. Furthermore, miR-24-3p in the Obese_Specific_Down category is known to be involved in both adipogenesis and the inflammatory response^38^.

### Identification of DEMs that are predicted to regulate inflammatory response genes

**Figure 5:**
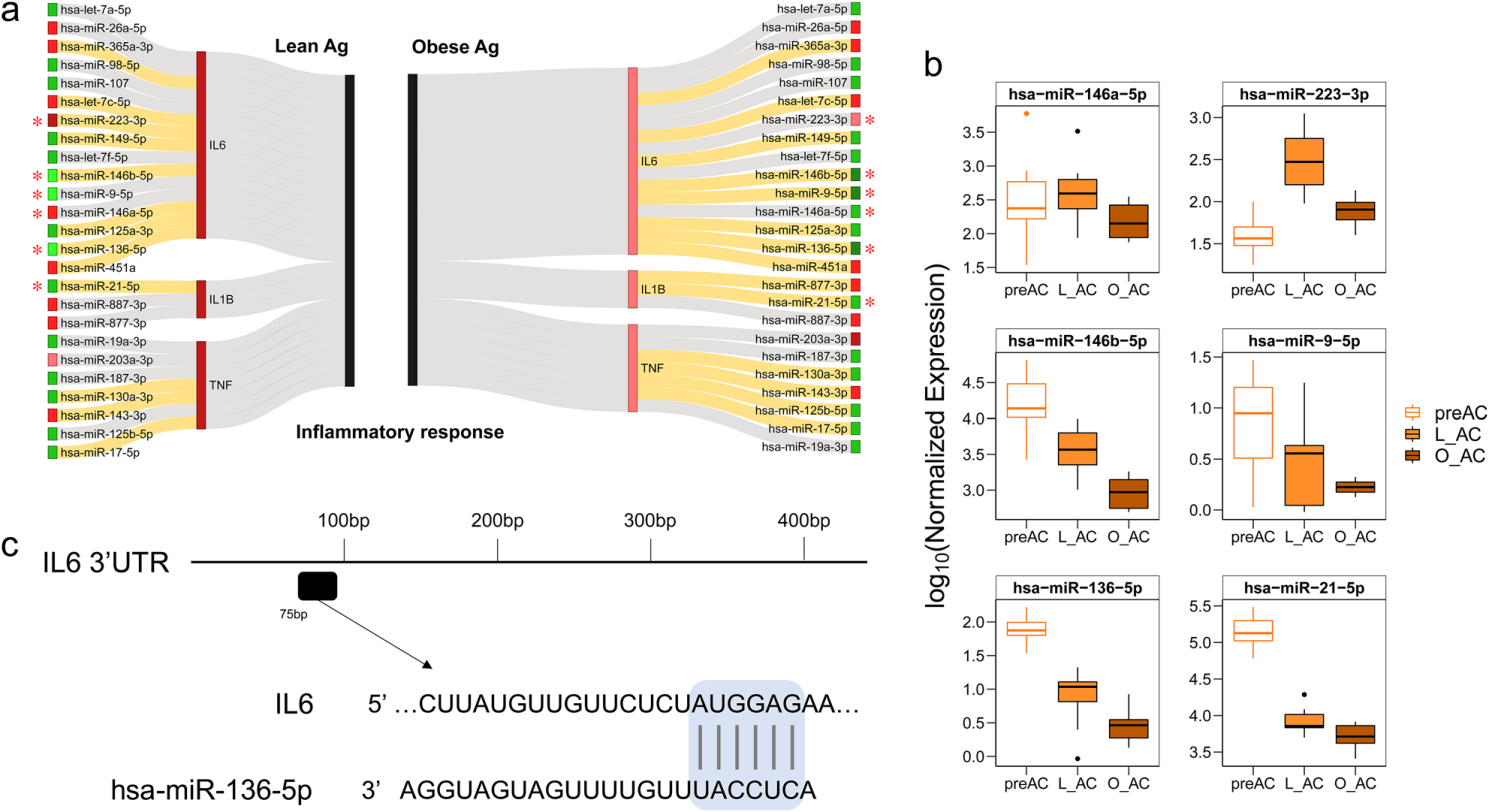
Analysis of DEMs that are preditcted to target inflammatory genes. (**a**) Sankey plot of miRNAs targeting inflammatory genes (*IL6, IL-1β*, and *TNF-α*). The miRNAs listed in the leftmost panel and rightmost panel are miRNAs that are predicted to target the three inflammatory genes identified from L_Ag and O_Ag, respectively. Downregulated and upregulated miRNAs are colored green and red, respectively. The depth of the colors reflects the differences in the absolute *log*_*2*_*FC* (i.e., |*log*_*2*_*FC|* between L_Ag and O_Ag. For instance, the |*log*_*2*_*FC|* of upregulated miR-223-3p is greater in L_Ag (dark red) than in O_Ag (light red) by more than 1; the |*log*_*2*_*FC|* of downregulated miR-146b-5p is greater (dark green) in O_Ag than in L_Ag (light green) by more than 1. In contrast, because the difference in the |*log*_*2*_*FC|* of let-7a-5p between L_Ag and O_Ag is less than 1, the same depth of color is used for both L_Ag and O_Ag). The miRNAs with an absolute *log*_*2*_*FC* difference of 1 or more between L_Ag and O_Ag are marked with asterisks. The miRNAs connected with the orange and gray lines are DEMs and non-DEMs, respectively. (**b**) Boxplots of the expression levels of miRNAs marked with asterisks in (**a**). Log10-transformed DESeq2-normalized expression levels (Y-axis) are plotted against each cell type (X-axis). (**c**) Example view of miR-136-5p binding sites predicted by TargetScan that targets *IL-6*. A diagram of the 3’ UTR of human *IL-6* was drawn based on RefSeq NM_000600.5.

Inflammatory genes are particularly interesting in the study of obesity because their upregulation in adipose tissue has long been implicated as a key aspect of obesity^33,40,41^. In our previous study, we demonstrated that inflammatory genes are even more upregulated in the lean condition than in the obese condition during adipogenesis, suggesting a positive role of inflammatory genes in adipogenesis. Therefore, we decided to examine how DEMs that are expected to target inflammatory response genes are altered during adipogenesis in lean and obese subjects (i.e., L_Ag *vs.* O_Ag). As shown in Fig. 5a, Sankey plot analysis connected a total of 25 miRNAs that are known to be regulators of canonical inflammatory genes involved in obesity, such as *TNF-α*, *IL-6*, and *IL-1β*, to the list of L_Ag-associated DEMs and O_Ag-associated DEMs. Note that all three inflammatory genes depicted in the Sankey plot were upregulated during adipogenesis in our previous study^33^ (represented in red bars). Notably, a larger number of miRNAs linked to these three upregulated inflammatory genes were downregulated than upregulated in both lean and obese conditions (Fig. 5a); 15/25 (60%, represented green boxes) and 16/25 (64%, represented green boxes) were downregulated miRNAs in L_Ag and O_Ag, respectively. The list of these miRNAs and their directions of up- and downregulation were mostly consistent between L_Ag and O_Ag; however, some miRNAs showed significantly different trends between lean and obese conditions (Fig. 5a). For instance, miR-223-3p was upregulated during adipogenesis in both conditions, but the level of upregulation in L_Ag was significantly higher than that in O_Ag. miR-146b-5p, miR-9-5p, miR-136-5p and miR-21-5p were downregulated during adipogenesis in both conditions, but the level of downregulation in O_Ag was significantly greater than that in L_Ag (Fig. 5b). Most strikingly, miR-146a-5p showed opposite expression changes: upregulation in L_Ag and downregulation in O_Ag (Fig. 5b). Interestingly, three of the 6 miRNAs in Fig. 5c were predicted to target at least one major inflammatory gene, *IL-6* or *IL1β*, with a score above 85 by TargetScan (release 8.0) (Additional file 3: Fig. S4), as depicted for miR-136-5p as an example (Fig. 5c).

### Comparison of adipogenesis-associated common DEMs to those of Ortega et al. (2010)

Ortega et al.^31^ identified adipogenesis-related miRNAs by investigating a total of 723 human and 76 viral mature miRNAs using human SAT samples from nonobese (n = 6) and obese (n = 9) samples. They identified a list of adipogenesis-related miRNAs subcategorizing into adipogenesis-promoting miRNAs and anti-adipogenesis miRNAs by investigating changes in gene expression during adipogenesis by comparing the gene expression of preACs (starting culture preACs at time 0 by following the differentiation protocol) to differentiated ACs (after the 7th day or 14th day of culture, where mature adipocytes started to be detected on the 7th day). We decided to validate our adipogenesis-associated miRNAs by comparing our list of miRNAs with that of Ortega et al.^31^ because the study design was similar to ours in that it compared gene expression between preACs and AC. Interestingly, as shown in Table S5 (Additional file 6: Table S5), the majority of miRNAs provided in Ortega et al.^31^ were found to be concordant with our DEMs even in terms of directional changes related to adipogenesis. In particular, the most remarkably downregulated genes in Ortega et al.^31^, including miR-503, miR-221, miR-424, miR-210, and miR-31, were all greatly downregulated in ACs compared to preACs in our study, and miR-30b and miR-30c, which were highly upregulated in the study by Ortega et al.^31^, were also upregulated in our study. In contrast, miR-143, miR-145, and miR-150 showed opposite expression trends in the two studies: they were antiadipogenesis factors in the study by Ortega et al.^31^ vs. adipogenesis-promoting in our study. Note that the findings regarding miR-143 have been contradictory in other studies described in the Introduction.

As a further validation, we decided to examine the presence of miRNA genes in the Mouse Genome Informatics (MGI) database (http://www.informatics.jax.org), a database that provides information about mouse mutant phenotypes. Indeed, we found that, among the DEMs identified in the present work, a total of 11 miRNAs have already been tested for their functional roles by examining mouse mutant phenotypes by introducing various types of mutations, including knockout, radiation or chemically induced mutagenesis (Additional file 7: Table S6). Of the 11 miRNAs, two miRNAs, miR-760 (Mir760 in mouse) and miR-184 (Mir184 in mouse), were found to have phenotypes associated with inflammation and obesity. In particular, transgenic insertion of Mir184 (a mouse ortholog of human miR-184), a downregulated miRNA in the present work (Additional file 2: Table S2), was reported to cause weight loss in mice, which strongly supports that miR-184 plays an important regulatory role in adipogenesis.

## Discussion

Numerous previous studies have attempted to identify obesity-related miRNAs using various study designs along with tissue samples and miRNA detection platforms, as outlined in Iacomino and Siani et al.^19^ and Ling et al.^42^. As expected, different obesity-associated miRNAs were identified in several studies, and the lists did not overlap well with each other, i.e., a miRNA that was found to promote adipogenesis in one study may have been found to trigger antiadipogenic effects in another study. For instance, for miR-221/222, Skarn et al.^32^ reported downregulation, whereas Parra et al.^43^ reported upregulation. Similarly, some studies showed that miR-143 is involved in promoting adipogenesis^25,42,44,45^, while others reported it to be an antiadipogenic miRNA^46^. According to Xie et al.^25^, the overlap between the Esau et al.^44^ and Kajimoto et al.^24^ studies was very small except for only one miRNA in the lists of adipocyte-important miRNAs.

Comparing gene expression between two different physiological conditions is a very popular approach in recent disease transcriptomics studies. However, it is still not easy to study changes in gene expression associated with obesity for several reasons. First, different adipose tissue types (i.e., VAT or SAT) have different gene expression patterns even in the same individual. Second, adipose tissue is comprised of a mixed population of several cell types, including ACs, preACs, endothelial cells, and macrophages^47–52^. Third, the interactions in miRNA-target mRNAs are highly complex and complicated, i.e., multiple miRNAs can target a single mRNA, or multiple mRNAs can share a single miRNA as a regulator^53–57^. Fourth, it is extremely difficult to obtain transcriptomes from healthy normal adipose tissue and cells purified from it to compare them to those from obese adipose tissue and cells. In fact, as discussed in our previous study, transcriptome-based studies designed to compare gene expression from purified ACs and preACs derived from VATs are extremely rare. Therefore, most of the studies were based on investigating solely miRNAs not involving mRNAs, and few studies have simultaneously investigated miRNAs and mRNAs using transcriptomes produced by microarrays or RNA-seq techniques. In that sense, the overlap of the list of DEMs between ours and Ortega et al.’s is quite remarkable (Additional file 6: Table S5), considering that the two studies used different tissue sources (cultured preAC and AC cells after isolation of SATs in Ortega et al.^31^ and purified AC and preAC from isolated human VATs in ours) and platform technologies (microarray in Ortega et al.^31^ and miRNA-seq in ours) to identify DEMs.

We demonstrated in the present work that, although several miRNAs, including miR-223-3p, 200a-3p, 200b-5p, 26b-3p, 26a-2-3p, 98-3p, 24-3p, and 675-5p, were altered differently during adipogenesis between lean and obese conditions, especially in terms of the magnitudes of expression changes (Fig. 4), adipogenesis in the two conditions mostly shared underlying molecular pathways controlled by miRNAs (Additional file 3: Fig. S1). We showed that L_Ag required greater upregulation of adipogenesis-promoting miRNAs than O_Ag, whereas O_Ag required greater downregulation of adipogenesis-inhibiting miRNAs than L_Ag, given that the upregulation and downregulation of a gene can be interpreted as promoting and inhibiting a specific physiological response, respectively (Figs. 3 and 4). All these results indicate that the same set of genes is involved in adipogenesis in both lean and obese conditions and that the fate toward either lean or obese status might be determined by a delicate balance maintained by the up- and downregulation of miRNAs controlling those genes, not by inducing or blocking the expression of an entirely different set of genes. Moreover, some of the miRNAs have been detected as circulating miRNAs that have been proposed as surrogate biomarkers of obesity^58,59^. For instance, several DEMs in the present work, including miR-144-5p, miR-192, miR-320, miR-378, miR-122, miR-24-3p, miR-223-3p and miR-146-5p, have been found to have strong correlations with various obesity-related metabolic indices in blood, such as serum leptin and triglycerides^60–64^. In addition, these circulating miRNAs are also reported to play significant regulatory roles in gene expression in other tissues and to influence many diseases such as metabolic syndromes and cancers^65,66^.

It is not easy to understand how the up- and downregulation of specific miRNAs, which are subsequently linked to the expression of target mRNAs associated with various cellular functions, are relevant to regulating adipogenesis because up- or downregulation of a single mRNA could be the combined outcome of various regulatory mechanisms, including multiple epigenetic regulators not only by miRNAs but also by other epigenetic changes such as chromatin modification or methylation that target the mRNA. In Fig. 5, we show that three important obesity-related genes, *IL6, IL-1β*, and *TNF-α*, are actually linked to multiple regulator DEMs altered during L_Ag and O_Ag. The three mRNAs were all significantly upregulated during adipogenesis (i.e., adipogenesis-promoting genes), although they were more highly upregulated in L_Ag than in O_Ag (represented by depth of red in the bars representing FCs of each of these genes in Fig. 5a). A total of 25 miRNAs (15 miRNAs for *IL-6*, 3 miRNAs for *IL-1β*, and 7 miRNAs for *TNF-α*) were found to target these three genes, and the directions of up- or downregulated miRNAs were mostly concordant between lean and obese conditions, except for one miRNA, miR-146a-5p (upregulated in L_Ag but downregulated in O_Ag). However, it is notable that the magnitudes of FCs of some miRNAs differed between L_Ag and O_Ag (Fig. 5b), so the level of miRNA expression alteration could ultimately lead to changes in the expression of the three mRNAs with distinct effects on adipogenesis in L_Ag and O_Ag.

## Conclusions

Overall, we confirmed not only that miRNAs are important epigenetic regulators controlling adipogenesis but also that L_Ag and O_Ag require some distinct miRNA perturbations, despite sharing many underlying molecular pathways. Our results will contribute to identifying entire regulatory circuits controlling obesity-related genes and developing strategies to effectively manage them to prevent problematic obesity.

## Methods

### Preparations of miRNA data from human adipocytes and preadipocyte samples

The methods for preparing AC and preAC samples and transcriptomes including miRNAs used in the present work were described in our previous study^33^. Basically, the data were produced by the Korea National Institute of Health (KNIH) in an effort to participate in the International Human Epigenome Consortium (IHEC). Data accessibility was granted by KNIH through strict regulations after evaluating the request by KNIH DAC (data access committee).

### Analysis of mRNA-seq data to identify DEGs

FastQC (https://www.bioinformatics.babraham.ac.uk/) was used to check the quality of and filter the raw sequence reads. The low-quality sequence reads and adaptor sequence reads were trimmed off by applying Trimmomatic (v0.35)^67^. After quality control, STAR (v2.7.1a)^68^ was then used to align the reads on the reference genome fasta file (GRCh38/hg38). For annotation, the release 30 human GTF file was downloaded from GENCODE (https://www.gencodegenes.org/), and the protein-coding genes were extracted. The mapped reads were quantified with HTSeq-count. DEG analysis was performed by DESeq2.

### Analysis of miRNA-seq data to identify DEMs

The raw sequence reads were filtered based on quality using FastQC (https://www.bioinformatics.babraham.ac.uk/), and the adaptor sequence reads and low-quality sequence reads were removed off by trimming using Cutadapt (v.2.1)^69^. miRDeep2 (https://www.mdc-berlin.de/content/mirdeep2-documentation) was then used to identify miRNAs by running ‘mapper.pl’ (generating a ‘fasta’ file that is used as the input to ‘quantifier.pl’ by merging sequencing reads with the same sequences) and ‘*quantifier.pl*’ (mapping sequencing reads by inputting the fasta file generated in the previous step and miRbase-driven mature miRNAs to miRbase-driven precursor miRNAs using ‘*bowtie*’). Read count was then estimated by DESeq2.

### miRNA-target mRNA identification

Information on miRNA-mRNA targets was obtained from ‘miRTarBase 8.0’ (http://mirtarbase.cuhk.edu.cn/)^70^, i.e., a database providing a total of 479,340 experimentally validated miRNA-target mRNA pairs for a total of 4,312 miRNAs. A total of 353 out of the 812 total DEMs obtained from L_Ag were matched to the miRTarBase list, revealing a total of 4367 interactions with 2037 mRNAs (i.e., 1,110 of the 2037 DEGs that were linked to 251 of the 353 DEMs), while a total of 331 out of the 748 DEMs obtained from O_Ag were matched to the miRTarBase list, revealing a total of 4820 interactions with 2,180 mRNAs (i.e., 1373 of the 2,180 that were linked to 246 of the 331 DEMs).

### miRNA binding site prediction

miRNA binding positions were predicted by TargetScan (release 8.0) (http://www.targetscan.org), which provides a robust prediction algorithm for the numerous predicted target pairs between miRNAs and genes by several measures.

### Data analysis

Statistical analyses and plotting experiments were performed using R (v3.5.1) with the Bioconductor 3.8 package. We performed GO analysis using the Database for Annotation, Visualization, and Integrated Discovery (DAVID) tool. PCA and unsupervised hierarchical clustering analysis were performed by the “FactoMineR” R package and the “stats” R package, respectively. The majority of the plots in this article were drawn using the “ggplot2” R package. The heatmap was plotted by the “gplots” R package. Other batch jobs were scripted with custom-built Python codes (https://www.python.org/).

### Ethics approval and consent to participate

This study was performed in accordance with the principles of the Declaration of Helsinki and was approved by the Kangwon National University Hospital (Chuncheon, Korea) Institutional Review Board (IRB) (KWNUIRB-2017-11-003).

## Supplementary Information

Supplementary Table S1.

Supplementary Table S2.

Supplementary Figures.

Supplementary Table S3.

Supplementary Table S4.

Supplementary Table S5.

Supplementary Table S6.

## Data Availability

Data are available in either of the two databases, IHEC (https://epigenomesportal.ca/ihec/index.html) or CODA (https://codo.nih.go.kr/index.do, CODA accession number: R00315).

## Abbreviations

preAC: Preadipocyte
AC: Adipocyte
SAT: Subcutaneous adipose tissue
L_Ag: Lean adipogenesis
O_Ag: Obese adipogenesis
VAT: Visceral adipose tissue
DEG: Differentially expressed gene
DEM: Differentially expressed miRNA
Ag_DEM: Adipogenesis-associated DEMs
PCA: Principal component analysis
GO: Gene ontology
FC: Fold change
L_AC: Lean adipocyte
O_AC: Obese adipocyte
