# Supplementary material for: Adipocyte differentiation between obese and lean conditions depends on changes in miRNA expression
Source: Sci Rep. 2022 Jul 7;12:11543. doi: 10.1038/s41598-022-15331-2 (PMC9262987; doi:10.1038/s41598-022-15331-2)
Supplement: Supplementary file 3 — Supplementary Figures. [file 41598_2022_15331_MOESM3_ESM.docx]

**Adipocyte differentiation between obese and lean conditions depends on changes in miRNA expression**

Yerim Heo^†^, Hyunjung Kim^†^, Jiwon Lim, Sun Shim Choi*

Division of Biomedical Convergence, College of Biomedical Science, Institute of Bioscience & Biotechnology, Kangwon National University, Chuncheon 24341, Korea

**Corresponding author:

Tel: +82-33-250-6487

E-mail: schoi@kangwon.ac.kr

^†^ These authors contributed equally to this work.


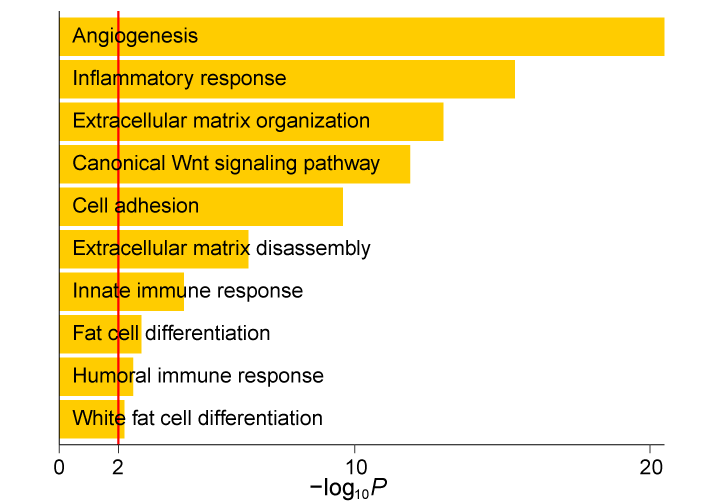


**Fig. S1 GO analysis of DEGs predicted to be regulated DEMs.**

After obtaining DEGs, miRNA-mRNA target information for the DEGs was obtained by matching the list of DEGs to the list of miRTarBase 8.0, as described in the Materials and Methods. The DEGs that were matched to the miRNAs in miRTarBase were only used for GO analysis. The top GO terms estimated based on the functional terms defined in the biological process category are depicted as bar graphs. Negative log10-transformed *P value*s (X-axis) against each functional term (Y-axis) are depicted as yellow bars, and the red line annotates significance at a *P value* of 0.01 (-log_10_*P* = 0.01).


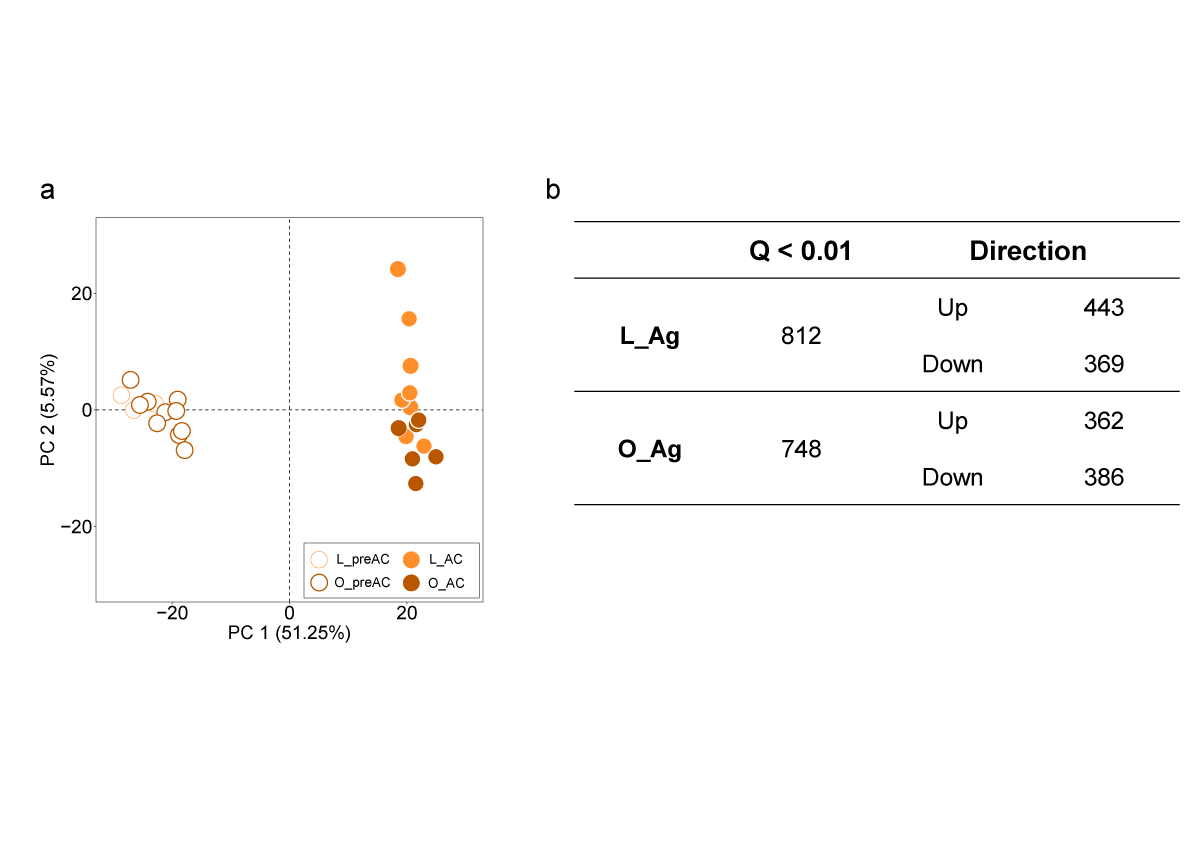


**Fig. S2 PCA and the numbers of DEMs estimated under L_Ag and O_Ag conditions.**

a. PCA of the same samples shown in Fig. 1c but lean or obese conditions of each sample are depicted. b. The numbers of upregulated and downregulated DEMs estimated in L_Ag and O_Ag, respectively.


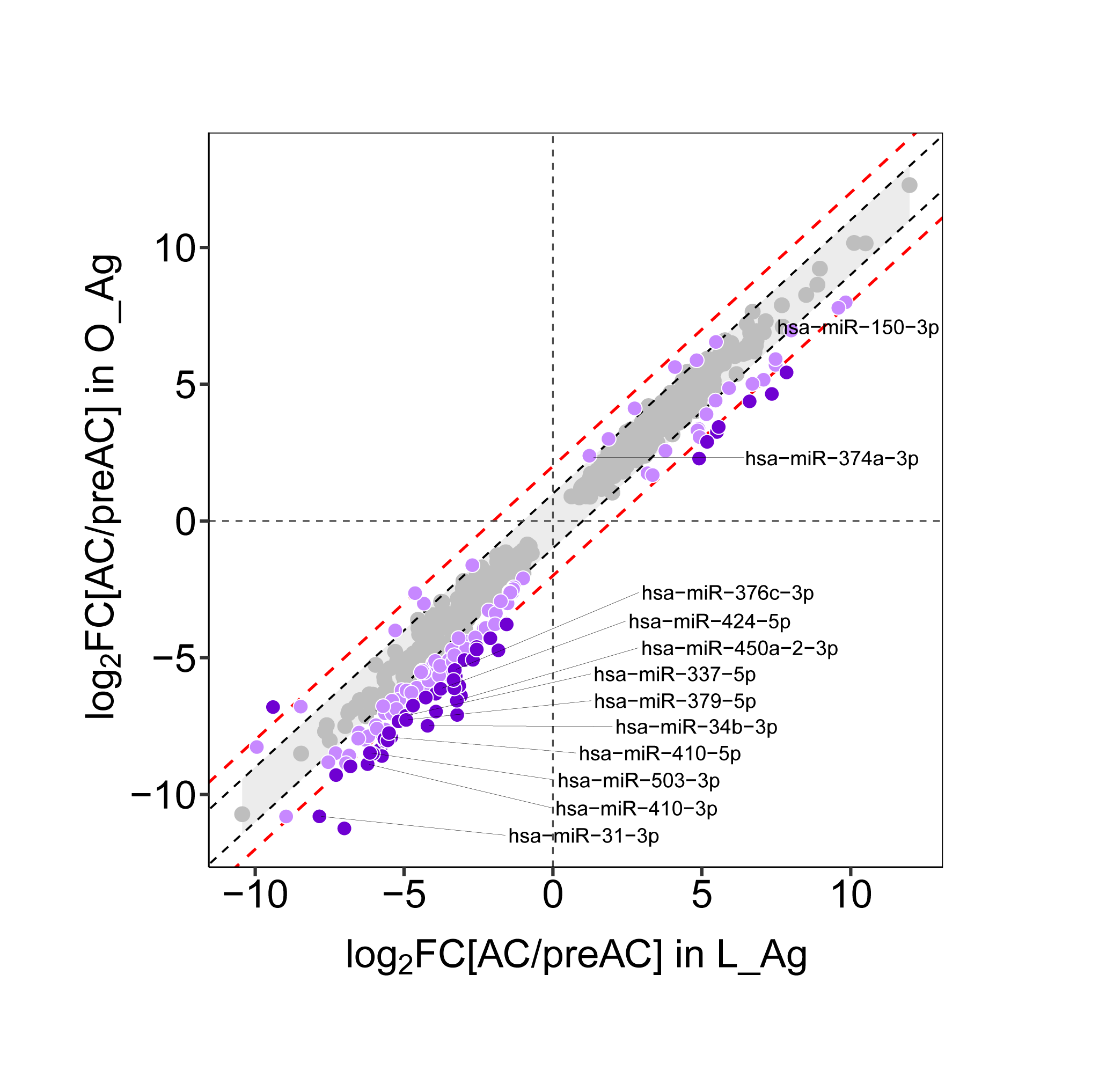


**Fig. S3 Scatter plot analysis of adipogenesis-associated FCs of each common DEM.**

*Log_2_FC* values of miRNA expression changes in L_Ag (X-axis) were plotted against those in O_Ag (Y-axis). The degree of difference in *Log_2_FC* values between L_Ag and O_Ag (i.e., ∆| *Log_2_FC*|) for each miRNA is represented in different colors (gray, ∆| *Log_2_FC*| < 1; light purple, 1≤∆| *Log_2_FC*|<2; dark purple, 2≤ ∆| *Log_2_FC*|). The black and red dashed lines represent the boundaries where the *Log_2_FC* values between L_Ag and O_Ag differ by 1 and 2, respectively (black, Y=X±1; red, Y=X±2). The miRNAs indicated by corresponding names are the miRNAs that overlap with the list of adipogenesis-associated miRNAs identified by Ortega et al. [31].


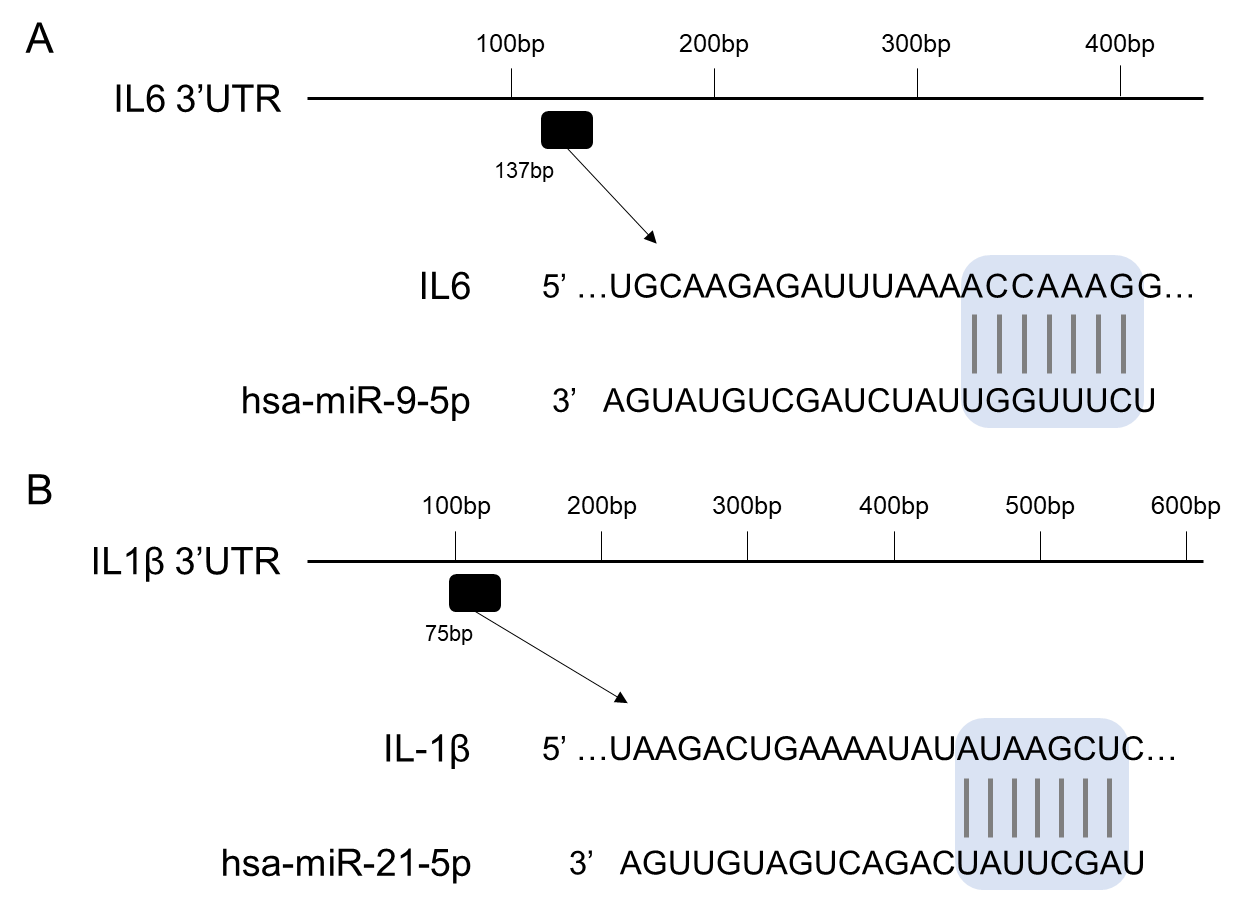


**Fig. S4 A depiction of the miR-9-5-p and miR-21-5p binding sites predicted by TargetScan.**

Diagrams of the 3’ UTRs of human *IL-6* and *IL-1β* were drawn based on NM_000600.5 and NM_000576.3, respectively.
