# Supplementary material for: Adipocyte differentiation between obese and lean conditions depends on changes in miRNA expression
Source: Sci Rep. 2022 Jul 7;12:11543. doi: 10.1038/s41598-022-15331-2 (PMC9262987; doi:10.1038/s41598-022-15331-2)
Supplement: Supplementary file 6 — Supplementary Table S5. [file 41598_2022_15331_MOESM6_ESM.docx]

**Table S5. Comparison of adipogenesis-associated miRNAs identified in the present work and Ortega et al.’s study.**

| **Ortega et al. (2010)** | |  | **Our results** | | | |  |
| --- | --- | --- | --- | --- | --- | --- | --- |
| **miRNA (P < 0.0001)** | **Direction** |  | **miRNA (Q < 0.01)** | **log2FoldChange** | | **Direction** | |
|  |  |  |  | **L_Ag** | **O_Ag** |  |  |
| hsa-let-7d | Up |  | hsa-let-7d-3p | 1.710 | 1.673 | Up | |
|  |  |  | hsa-let-7d-5p | 1.615 | 1.735 | Up | |
| hsa-let-7g | Up |  | hsa-let-7g-3p | 2.157 | 2.053 | Up | |
|  |  |  | hsa-let-7g-5p | 2.250 | 2.142 | Up | |
| hsa-miR-100 | Down |  | hsa-miR-100-5p | -1.525 | -3.009 | Down | |
|  |  |  | hsa-miR-100-3p | -5.956 | -5.257 | Down | |
| hsa-miR-101 | Up |  | hsa-miR-101-3p | 1.208 | N.S. | Up | |
|  |  |  | hsa-miR-101-5p | 1.815 | N.S. | Up | |
| hsa-miR-125b | Down |  | hsa-miR-125b-5p | N.S. | -1.257 | Down | |
|  |  |  | hsa-miR-125b-1-3p | -3.940 | -3.749 | Down | |
| hsa-miR-127-3p | Down |  | hsa-miR-127-3p | -5.063 | -6.989 | Down | |
| hsa-miR-130a | Down |  | hsa-miR-130a-3p | -1.747 | -2.307 | Down | |
|  |  |  | hsa-miR-130a-5p | -2.563 | -1.972 | Down | |
| hsa-miR-130b | Down |  | hsa-miR-130b-5p | -3.176 | -3.646 | Down | |
|  |  |  | hsa-miR-130b-3p | -4.162 | -4.561 | Down | |
| hsa-miR-136 | Down |  | hsa-miR-136-3p | -4.301 | -5.861 | Down | |
|  |  |  | hsa-miR-136-5p | -2.911 | -4.402 | Down | |
| hsa-miR-140-3p | Down |  | hsa-miR-140-3p | N.S. | -0.931 | Down | |
| hsa-miR-143 | Down |  | hsa-miR-143-5p | 3.717 | 4.054 | Up | |
|  |  |  | hsa-miR-143-3p | 2.473 | 2.012 | Up | |
| hsa-miR-145 | Down |  | hsa-miR-145-3p | 3.240 | 2.679 | Up | |
|  |  |  | hsa-miR-145-5p | 4.757 | 4.744 | Up | |
| hsa-miR-148a | Up |  | hsa-miR-148a-3p | 1.250 | N.S. | Up | |
| hsa-miR-150-3p | Down |  | hsa-miR-150-3p | 9.832 | 7.996 | Up | |
| hsa-miR-15a | Up |  | hsa-miR-15a-5p | N.S. | -0.775 | Down | |
| hsa-miR-15b | Down |  | hsa-miR-15b-5p | -1.393 | -1.613 | Down | |
|  |  |  | hsa-miR-15b-3p | -4.264 | -6.200 | Down | |
| hsa-miR-16 | Up |  | hsa-miR-16-5p | -1.260 | -1.781 | Down | |
|  |  |  | hsa-miR-16-2-3p | -2.232 | -2.502 | Down | |
| hsa-miR-185 | Up |  | hsa-miR-185-3p | N.S. | 0.771 | Up | |
| hsa-miR-196a | Up |  | hsa-miR-196a-5p | -2.427 | N.S. | Down | |
| hsa-miR-21-3p | Down |  | hsa-miR-21-3p | -4.021 | -5.206 | Down | |
| hsa-miR-210 | Down(-4.3-fold) |  | hsa-miR-210-5p | -3.024 | -2.464 | Down | |
|  |  |  | hsa-miR-210-3p | -2.958 | -2.842 | Down | |
| hsa-miR-214 | Down |  | hsa-miR-214-5p | -1.799 | -2.069 | Down | |
|  |  |  | hsa-miR-214-3p | -0.953 | -1.201 | Down | |
| hsa-miR-218 | Up |  | hsa-miR-218-5p | -2.384 | -2.346 | Down | |
|  |  |  | hsa-miR-218-2-3p | 1.664 | 2.105 | Up | |
|  |  |  | hsa-miR-218-1-3p | -1.770 | -1.706 | Down | |
| hsa-miR-22 | Up |  | hsa-miR-22-5p | 1.601 | 1.566 | Up | |
| hsa-miR-221-5p | Down(-4.9-fold) |  | hsa-miR-221-5p | -6.512 | -7.731 | Down | |
| hsa-miR-221-3p | Down |  | hsa-miR-221-3p | -5.080 | -6.180 | Down | |
| **Table S5. Continued** | | | | | | | |
| hsa-miR-24 | Down |  | hsa-miR-24-3p | N.S. | -0.843 | Down | |
|  |  |  | hsa-miR-24-1-5p | 1.465 | 1.217 | Up | |
|  |  |  | hsa-miR-24-2-5p | -1.803 | -1.357 | Down | |
| hsa-miR-27a | Down |  | hsa-miR-27a-5p | -1.798 | -1.684 | Down | |
|  |  |  | hsa-miR-27a-3p | -1.145 | -1.202 | Down | |
| hsa-miR-27b | Down |  | hsa-miR-27b-5p | N.S. | -1.255 | Down | |
| hsa-miR-30a-5p | Up(4.0-fold) |  | hsa-miR-30a-5p | -1.200 | -1.552 | Down | |
| hsa-miR-30b | Up(3.1-fold) |  | hsa-miR-30b-5p | 1.655 | 1.193 | Up | |
|  |  |  | hsa-miR-30b-3p | 1.679 | 1.804 | Up | |
| hsa-miR-30c | Up(5.1-fold) |  | hsa-miR-30c-5p | 0.953 | N.S. | Up | |
|  |  |  | hsa-miR-30c-2-3p | 1.283 | 1.197 | Up | |
|  |  |  | hsa-miR-30c-1-3p | 0.886 | 0.865 | Up | |
| hsa-miR-31-5p | Down |  | hsa-miR-31-5p | -7.297 | -8.482 | Down | |
| hsa-miR-31-3p | Down(-2.6-fold) |  | hsa-miR-31-3p | -7.842 | -10.795 | Down | |
| hsa-miR-324-5p | Up |  | hsa-miR-324-5p | 1.049 | 1.108 | Up | |
| hsa-miR-337-5p | Down |  | hsa-miR-337-5p | -4.938 | -7.120 | Down | |
| hsa-miR-34a | Up(2.5-fold) |  | hsa-miR-34a-5p | -2.854 | -3.208 | Down | |
|  |  |  | hsa-miR-34a-3p | -3.671 | -3.896 | Down | |
| hsa-miR-34b-3p | Up |  | hsa-miR-34b-3p | -4.211 | -7.484 | Down | |
| hsa-miR-374a | Up |  | hsa-miR-374a-3p | 1.226 | 2.388 | Up | |
| hsa-miR-376a | Down |  | hsa-miR-376a-3p | -3.719 | -5.215 | Down | |
|  |  |  | hsa-miR-376a-5p | -4.013 | -5.245 | Down | |
|  |  |  | hsa-miR-376a-2-5p | -4.441 | -4.960 | Down | |
| hsa-miR-376c | Down |  | hsa-miR-376c-3p | -3.297 | -5.448 | Down | |
| hsa-miR-377 | Down |  | hsa-miR-377-3p | -4.881 | -6.546 | Down | |
|  |  |  | hsa-miR-377-5p | -3.953 | -5.114 | Down | |
| hsa-miR-379 | Down |  | hsa-miR-379-3p | -6.843 | -8.571 | Down | |
|  |  |  | hsa-miR-379-5p | -4.926 | -7.270 | Down | |
| hsa-miR-381 | Down |  | hsa-miR-381-5p | -4.415 | -6.114 | Down | |
|  |  |  | hsa-miR-381-3p | -5.397 | -6.930 | Down | |
| hsa-miR-409-3p | Down |  | hsa-miR-409-3p | -6.323 | -7.993 | Down | |
| hsa-miR-410 | Down |  | hsa-miR-410-5p | -5.435 | -7.904 | Down | |
|  |  |  | hsa-miR-410-3p | -6.226 | -8.885 | Down | |
| hsa-miR-424 | Down(-4.6-fold) |  | hsa-miR-424-5p | -3.767 | -6.132 | Down | |
|  |  |  | hsa-miR-424-3p | -5.295 | -6.897 | Down | |
| hsa-miR-450a | Down |  | hsa-miR-450a-5p | -3.610 | -5.594 | Down | |
|  |  |  | hsa-miR-450a-2-3p | -3.225 | -6.568 | Down | |
| hsa-miR-452 | Up |  | hsa-miR-452-5p | 2.594 | 2.413 | Up | |
| hsa-miR-494 | Down |  | hsa-miR-494-3p | -5.587 | -6.851 | Down | |
|  |  |  | hsa-miR-494-5p | -6.200 | -7.867 | Down | |
| hsa-miR-503 | Down(-6.7-fold) |  | hsa-miR-503-3p | -6.145 | -8.476 | Down | |
|  |  |  | hsa-miR-503-5p | -5.637 | -7.027 | Down | |
| hsa-miR-532-5p | Up |  | hsa-miR-532-5p | -1.470 | -1.968 | Down | |
| hsa-miR-542-3p | Down |  | hsa-miR-542-3p | -3.364 | -5.139 | Down | |

N.S.=not significant (q > 0.01).
