# Supplementary material for: Adipocyte differentiation between obese and lean conditions depends on changes in miRNA expression
Source: Sci Rep. 2022 Jul 7;12:11543. doi: 10.1038/s41598-022-15331-2 (PMC9262987; doi:10.1038/s41598-022-15331-2)
Supplement: Supplementary file 7 — Supplementary Table S6. [file 41598_2022_15331_MOESM7_ESM.docx]

**Table S6. List of DEMs validated with mouse mutant phenotypes of MGI**

| miRNA | Orthologous Mouse Gene Symbol | Type of Mutation | Allele Symbol Gene; Allele Name | Strain State | Phenotypes (or Diseases) * | Phenotypes related to Obesity or Inflammation |
| --- | --- | --- | --- | --- | --- | --- |
| has-miR-1179 | Mir1193 | Knockout | Mirc6tm1.1Mzhu | ES cell | C, M |  |
|  |  |  |  |  |  |  |
|  |  | Knockout | Mirc36tm1.1Jcav | Sperm | B, E, G, H, L, A |  |
| has-miR-1291 | Mir1291 | Radiation induced | Eh | Embryo | B, CF, D, G, HE, H, I, A, R |  |
|  |  | Radiation induced | In(15)4H | Embryo | CF, G, HE, I, R, S, V; HTC1 |  |
|  |  | Chemically induced | In(15)35Rk | Embryo | R |  |
| has-miR-133b | Mir133b | Targeted | Mir133btm1.1Gfng | - | - |  |
|  |  | Knockout | Mir133btm1.2Gfng | - | - |  |
|  |  | Knockout | Mirc37tm1Boet | - | - |  |
|  |  | Knockout | Mirc37tm1Gvdz | - | - |  |
| has-miR-326 | Mir326 | Chemically induced | In(7)13Rk | Embryo/Sperm | R |  |
|  |  |  |  |  |  |  |
|  |  | Radiation induced | In(7Oca2;7Sox6)100H | - | CF, G, HP, L, A, M |  |
|  |  | Knockout | Mir326tm1Wtsi | - | - |  |
| has-miR-451a | Mir451a | Chemically induced | In(11)20Rk | Embryo | R |  |
|  |  | Targeted | In(11Trp53;11Wnt3)8Brd | Sperm | A |  |
|  |  | Knockout | Mir144/Mir451atm1.2Doca | Embryo | HP, H, IM, A |  |
|  |  | Knockout | Mir451atm1.1Mjwe | - | C, HP, H, IM |  |
|  |  | Knockout | Mir451atm1.2Doca | Embryo/Sperm | HP, IM |  |
|  |  | Knockout | Mir451atm1Eno | - | HP, H, IM |  |
|  |  | Targeted | Mir144/Mir451atm1.1Doca | - | - |  |
|  |  | Targeted | Mir451atm1.1Doca | - | - |  |
| has-miR-760 | Mir760 | Chemically induced | In(3)11Rk | Embryo | R |  |
|  |  | Radiation induced | Uoxin | Embryo/Sperm | H, IM(IF), A, RU; HU, K | ● |
| has-miR-1197 | Mir1197 | Knockout | Del(12Mir379-Mir410)2Arte | - | - |  |
|  |  | Knockout | Mirc6tm1.1Mzhu | - | C, M |  |
|  |  | Knockout | Mirc36tm1.1Jcav | Sperm | B, E, G, H, L, A |  |
| has-miR-184 | Mir184 | Chemically induced | In(9)26Rk | Embryo | R |  |
|  |  | Knockout | Mir184tm1Ics | - | C, E, H |  |
|  |  | Transgenic insertion | Tg(Ins2-Mir184)4Mnpy | - | C, E, G(W), H | ● |
|  |  | Transgenic insertion | Tg(Ins2-Mir184)96Mnpy | - | E, H |  |
| has-miR-449a | Mir449a | Knockout | Mir449aem1Xzhg | - | - |  |
|  |  | Knockout | Mir449aem2Xzhg | - | - |  |
|  |  | Knockout | Mir449atm1.1Doca | - | - |  |
|  |  | Knockout | Mirc34em1Jnch | - | - |  |
|  |  | Knockout | Mirc34tm1.1Mkss | - | - |  |
| has-miR-496 | Mir496a | Knockout | Del(12Mir379-Mir410)2Arte | - | - |  |
|  |  | Knockout | Mirc36tm1.1Jcav | Sperm | B, E, G, H, L, A |  |
|  |  | Knockout | Mir496atm1Wtsi | - | - |  |
| has-miR-543 | Mir543 | Knockout | Del(12Mir379-Mir410)2Arte | - | - |  |
|  |  | Knockout | Mirc6tm1.1Mzhu | - | C, M |  |
|  |  | Knockout | Mirc36tm1.1Jcav | Sperm | B, E, G, H, L, A |  |
| *** Abbreviations:** C, cellular; M, muscle; B, behavior; E, endocrine/exocrine; G, growth/size/body; H, homeostasis; L, liver/biliary; A, mortality/aging; CF, craniofacial; D, digestive/alimentary; HE, hearing/vestibular/ear; I, integument; R, respiratory; S, skeleton; V, vision/eye; HTC1, Ambras type hypertrichosis universalis congenita; HP, hematopoietic; IM, immune; IM(IF), immune (chronic inflammation); RU, renal/urinary; HU, hyperuricemia; K, kidney disease; G(W), growth/size/body (weight loss) | | | | | | |
